# Does the systemic administration of l-arginine affect dental implant stability in nicotine consumer dogs?

**DOI:** 10.1186/s40902-021-00292-9

**Published:** 2021-02-10

**Authors:** Bijan Movahedian, Mansour Rismanchian, Hooman Navaei, Saeid Tavanafar, Soheil Koushaei

**Affiliations:** 1 Department of Oral and Maxillofacial Surgery, School of Dentistry, Isfahan University of Medical Sciences, Isfahan, Iran; 2 Department of Prosthodontics, Dental Implants Research Center, School of Dentistry, Isfahan University of Medical Sciences, Isfahan, Iran; 3 Department of Oral and Maxillofacial Surgery, School of Dentistry, Isfahan University of Medical Sciences, Esfahan, Iran; 4 Department of Oral and Maxillofacial Surgery, School of Dentistry, Shiraz University of Medical Sciences, Shiraz, Iran; 5 Department of Oral and Maxillofacial Surgery, School of Dentistry, Qazvin University of Medical Sciences, Qazvin, Iran

**Keywords:** Implant stability quotient (ISQ), l-arginine, Nicotine, Osseointegration, Resonance frequency assessment (RFA)

## Abstract

**Background:**

Nicotine can have detrimental effects on dental implant osseointegration. This study aimed to evaluate the influence of systemic l-arginine supplement on the osseointegration of dental implants in nicotine consumer dogs.

**Methods:**

Twelve 1-year Labrador Retriever dogs had their right and left third and fourth mandibular premolars removed, and the sockets were left to heal for 6 months. Dogs were randomly divided into three groups (*n* = 16): group 1—0.2 mg/kg nicotine was injected twice daily; group 2—0.2 mg/kg nicotine was injected twice daily in addition to 200 mg/kg l-arginine capsules taken orally; and group 3—placebo. Forty-eight dental implants were inserted into the healed sockets of the dog’s mandible and were assessed by implant stability quotient (ISQ) using resonance frequency analysis (RFA) during 4 weeks and insertion and removal torque value analysis.

**Results:**

No implant failure occurred during the study period. The change in torque value between insertion and removal was similar in the placebo and nicotine+arginine consumer dogs (*p* = 0.276), which shows a positive effect of arginine supplementation in nicotine consumers. There was a significant difference in torque value change between nicotine+arginine vs. nicotine consumers (*p* = 0.049) and placebo vs. nicotine (*p* = 0.003). After 4 weeks, the placebo had the most significant improvement in torque value (47.0 ± 16.9), followed by nicotine+arginine (25.1 ± 37.8), and the worst torque value was for the nicotine group (− 5.7 ± 24.0) pound per inch. The results show that except in the first week, there are significant differences in ISQ between the groups in different periods. ISQ in all of the groups has reduced at first but then increased over time. At the time of implant placement, insertion torque was significantly higher in the nicotine consumer group than the nicotine+arginine consumer group and placebo group (*p* = 0.020).

**Conclusion:**

Arginine supplementation promotes bone healing and implant primary stability by improving dental implant osseointegration biomechanical characteristics.

## Background

One of the critical factors in dental implant treatment success rate is mechanical stability after surgical insertion and functional masticatory loading [1–6]. The surrounding bone tissue after preparation of the implant site sustains severe inevitable damage. The injury to the surrounding bony tissue triggers active bone remodeling to form new bone neighboring the dental implants during the healing period [4].

One of the significant bone formation indicators and remodeling is the osteoblastic activity, required in dental implant osteointegration and healing of fractured bone. Previous reports show that several amino acids play a beneficial role in bone formation. Arginine, a semi-essential amino acid, affects polyamine synthesis, stimulates growth hormone production, and improves endothelial function by enhancing nitric oxide formation [7–10].

Several risk factors, such as low quality and inadequate quantity of peri-implant bone, or various medical conditions, such as uncontrolled diabetes or osteoporosis, may endanger dental implant osteointegration and survival [11]. Another notable risk factor is tobacco smoking, attributed to dental implant failure [12]. Many authors studied tobacco smoking’s significance in peri-implant tissues and eventual implant failure [13–16]. The deleterious effect of tobacco smoking is mostly attributed to its nicotine content, although more than 4000 potentially toxic substances have been identified [17]. Nicotine is known to cause vasoconstriction and inhibit cellular healing responses [18]. Therefore, nicotine may harm dental implant osseointegration [19].

In the present study, we aimed to evaluate the effects of nicotine alone and in combination with arginine supplementation on early implant osseointegration.

## Methods

### Sample size estimation

We used previous trials, sixteen samples in each group required to reach a significant difference. Therefore, four dogs were designated in each group, and four implants for each dog have a 0.80 probability of having differences of *d* = 7.8 when ∝ = 0.05.

### Ethical considerations for using animals

All of the ethical issues concerning using the animals in a clinical trial by our university were followed. The ethical committee review board has confirmed the study protocol (IR.MUI.RESEARCH.REC.1398.005), and the reporting of the present study follows the ARRIVE checklist for all relevant items. All dogs were provided by the Isfahan University of Medical Sciences Animal Laboratory. We have used the minimum number of dogs required for this study, and all of the dogs survived after the study is finished.

### Experimental procedure

**Fig. 1:**
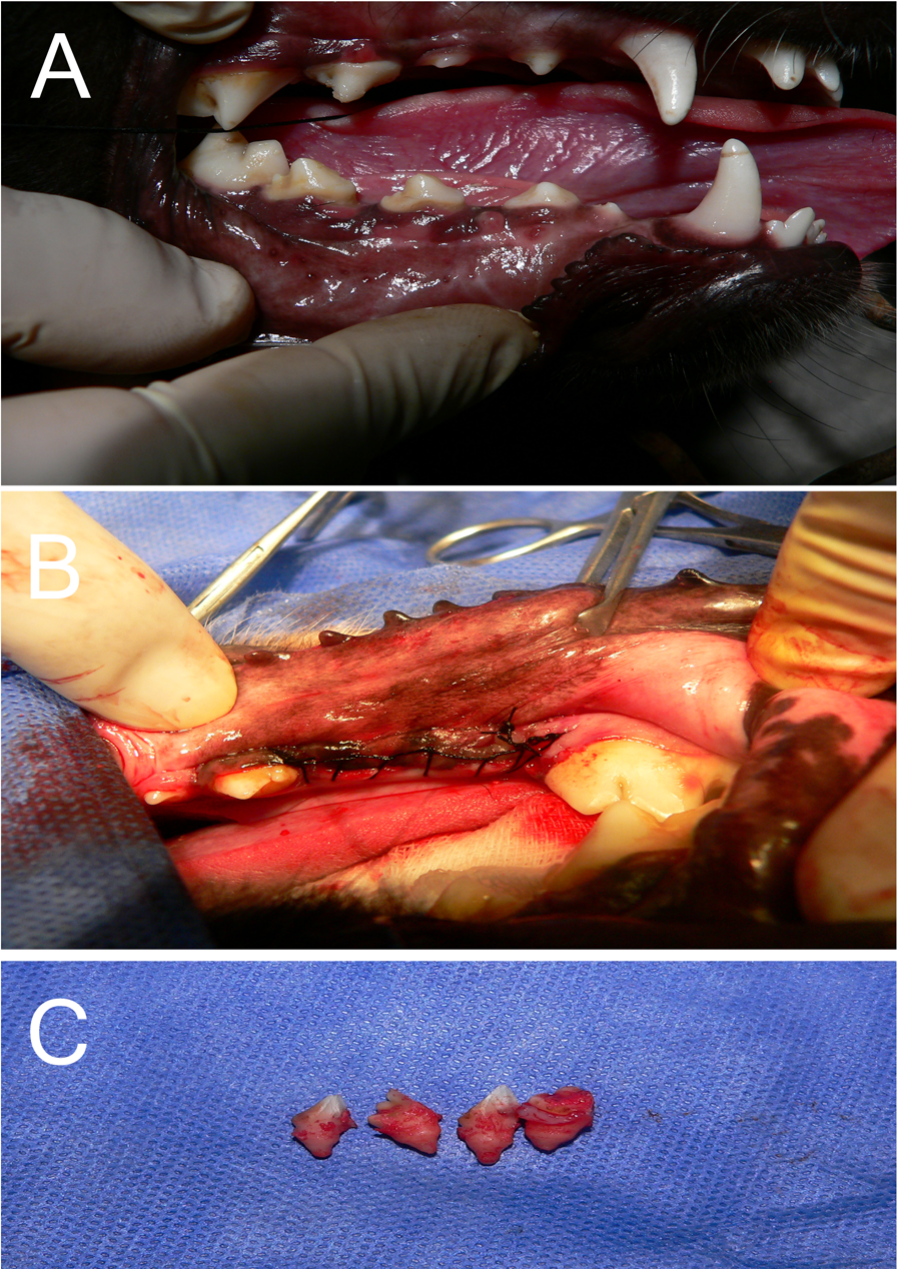
**a** Preoperative mandibular premolars. **b** Surgically removed mandibular premolars and **c** removed dogs’ teeth

Twelve 1-year-old male Labrador Retriever dogs were chosen for this study. Each dog, weighing approximately 30 kg, had healthy dentition and periodontium. During the experiment, the animals were housed in private kennels with adequate ventilation and fed a soft diet. Each dog was anesthetized by giving 5 mg/kg IV ketamine hydrochloride, followed by halothane and nitrous oxide inhalation. After sterile prep and draping, the dog mouth was cleaned with 0.2% chlorhexidine for 30 s. The throat pack using sterile gauze was placed around the endotracheal tube. Two percent lidocaine with 1:80,000 epinephrine was infiltrated on the buccal and lingual sides of the first and second mandibular premolars. Then, each tooth was sectioned and removed atraumatically (Fig. 1). Thus, a total number of four teeth in each dog were extracted for future implant placement. To ensure no remaining roots and to assess the teeth sockets, postoperative radiographs were obtained.

**Fig. 2:**
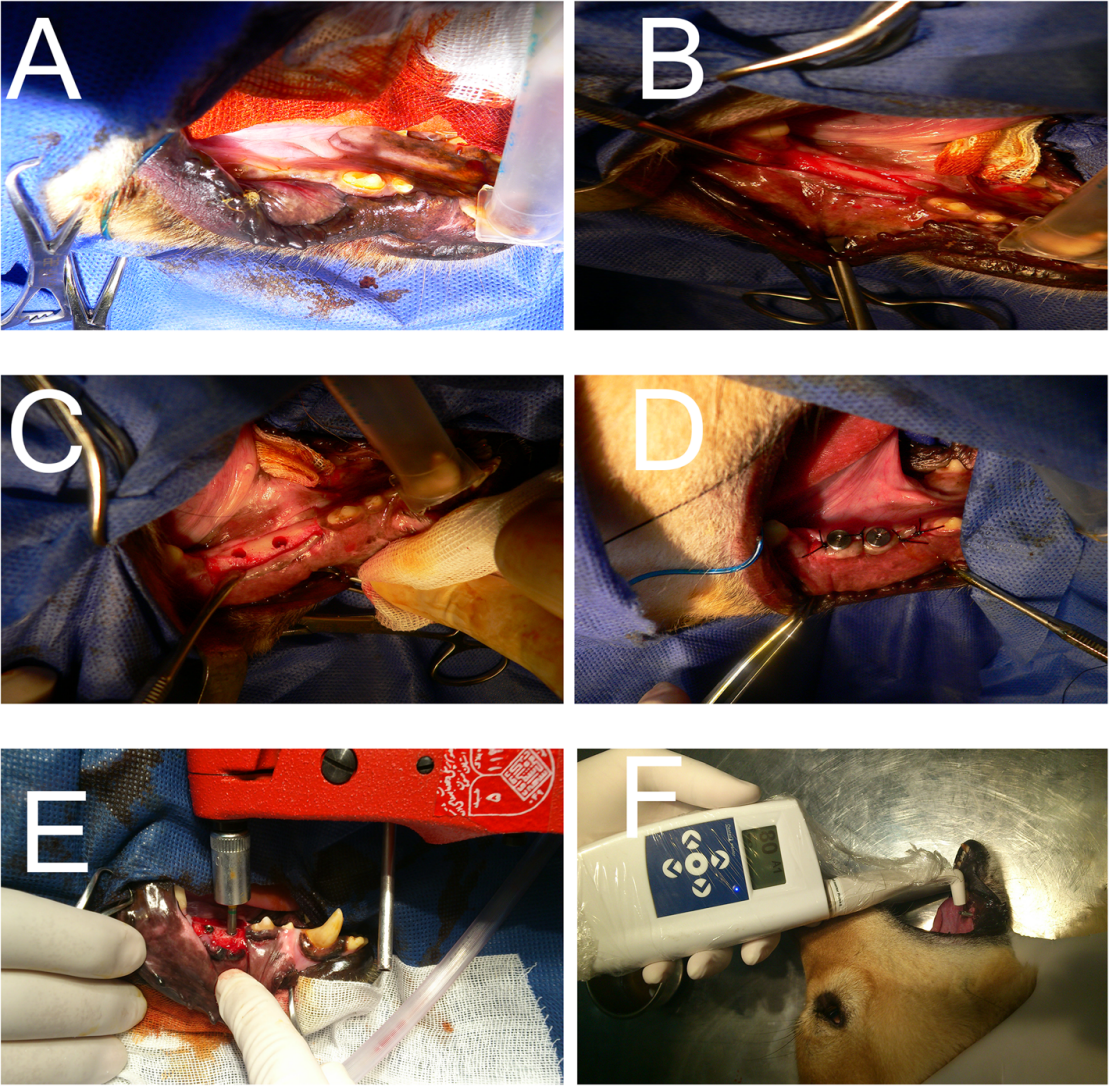
Clinical view after 6 months of healing. Surgical exposure of the previous extraction site. Implant osteotomy preparation and implant placement. Torque value measurement and ISQ measurement

Six months after the tooth removal, the dog’s mandible was examined clinically and radiographically, ready for implant placement (Fig. 2). Following the same anesthetic procedure, each dog was prepared for implant placement. After preparation and sterile draping of the surgical field, surgical blade number 15 was used to make a crestal incision, and a mucoperiosteal flap was reflected (Fig. 2). Dental implants were inserted using the manufacturer’s guidelines (3.8 mm × 10 mm, Bio Horizons Implant System Inc., Birmingham, AL, USA, Fig. 2). During dental implant insertion, the required torque was measured and recorded in pound per inch using a Dial Measuring Torque Wrenches (Torqueleader, Gedore, Bramley, Guildford, UK) (Fig. 2). The insertion of dental implants continued until reaching the level of the crestal bone. The polished parts were not inserted into the bone. Smart peg of Ostell (Osstell, Integration Diagnostics Ltd., Gothenburg, Sweden) was inserted into the dental implants. Resonance frequency analysis (RFA) values were measured by the Osstell device (Fig. 2). This measures the implant stability quotient (ISQ) unit value shown as a scale of reading on the device screen. A periapical radiograph was taken to ensure the proper position of dental implants to the adjacent bone.

Dogs were placed on a soft diet for 1 week. Each group consisted of four dogs with four implants. Dogs were randomly assigned to three groups;

*Nicotine-arginine group (n = 16)*: dogs in this group received 2 mg/kg/12 h nicotine subcutaneously and 200 mg/kg/daily l-arginine by prefabricated capsules in their daily foods.

*Nicotine group (n = 16)*: dogs in this group received 2 mg/kg/12 h nicotine subcutaneously and prefabricated empty capsules.

*Placebo (n = 16)*: dogs in this group were receiving the normal saline while injecting nicotine to other dogs and prefabricated empty capsules in their daily foods.

ISQ was recorded at the time of implant insertion and every week for 4 weeks. Except for the first and last ISQ recordings, which were under general anesthesia, other recordings were under mild sedation of dogs (Fig. 2). During the 4-week experimental period, an experienced veterinary visited the dogs twice daily to ensure their health. After the fourth week, dogs were put under general anesthesia, and the fifth ISQ were recorded, and the reverse torque of the implant was measured using a manual torque wrench. Laboratory assistants recorded all of the measurements and were blinded to the group allocations of the study.

### Statistical analysis

Data were presented as mean ± SD for continuous variables. In this analysis, one-way analysis of variance (ANOVA) with Tukey post hoc test, repeated measure (RE) ANOVA, and paired *t* test with two sides at the 5% level of significance were used. All the analyses were performed using the Statistical Package for Social Sciences version 16.0 (SPSS Inc., Chicago, IL, USA).

## Results

**Fig. 3:**
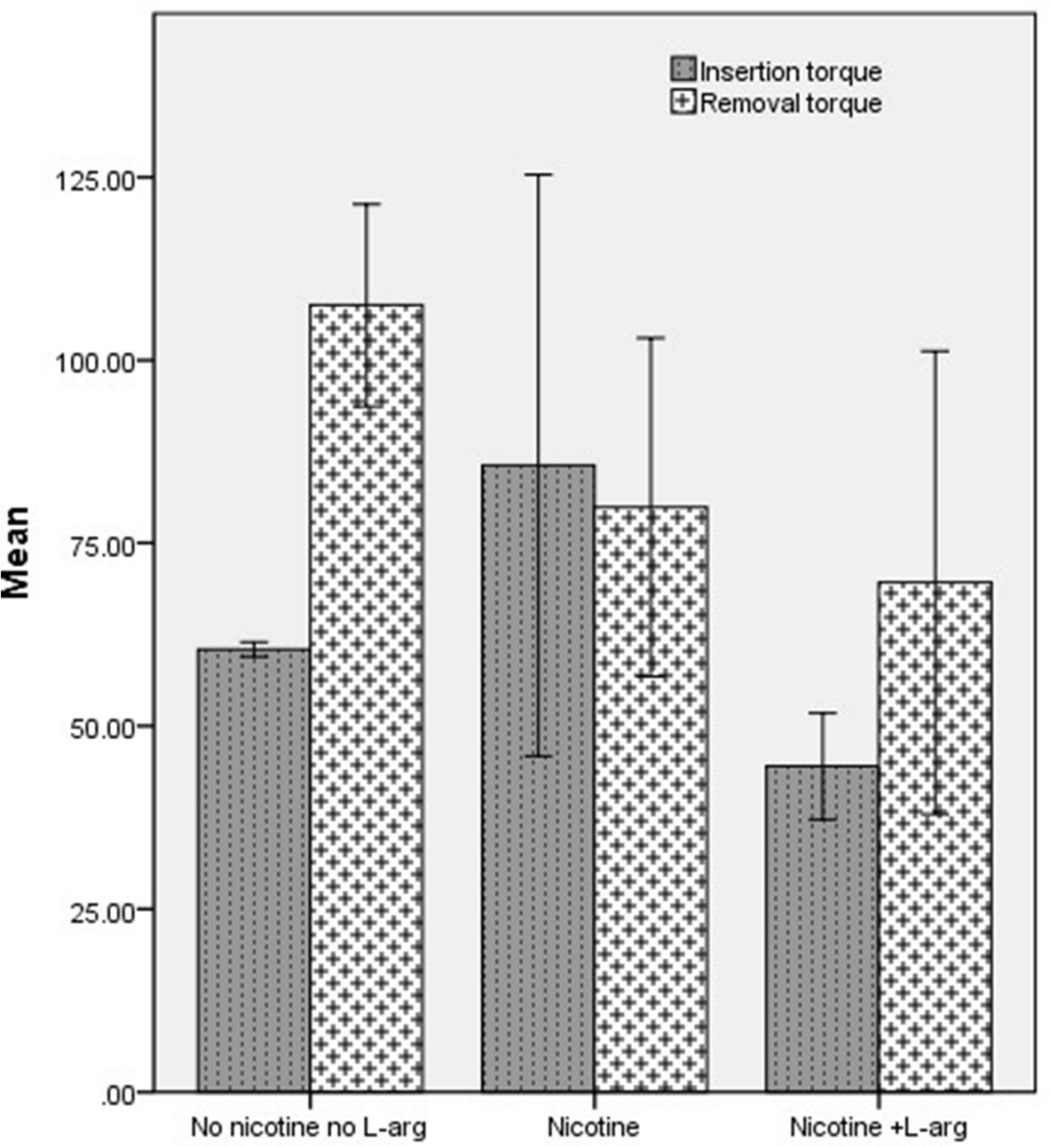
The mean of torque at insertion and removal time in the three groups

In this study, twelve dogs, each with four dental implants, were studied over 4 weeks. No implant failure occurred during the study. Torque value analysis is presented in Table 1. At the time of implant placement, insertion torque was significantly higher in the nicotine consumer group than the nicotine+arginine consumer group and placebo group (*p* = 0.020). After 4 weeks, dental implants’ removal torque was significantly different between nicotine+arginine consumer dogs and placebo, and placebo had higher removal torque (*p* = 0.039). When comparing insertion torques and removal torques of all the three groups, only the placebo group was significantly different (*p* < 0.001). Nicotine consumer dogs had also gained less torque upon removal than the other two groups during the 4 weeks. The other two groups had increased torque during the study period. Although there were no significant differences between nicotine+arginine consumers and placebo, placebo had higher torque values in two periods of time, which shows adverse effects of nicotine on implant torque, and using arginine might have compensated for adverse effects of nicotine. However, still, it is not comparable to when no nicotine is used (Fig. 3).

**Table 1:** Comparison of torque between the 3 groups (pound per inch)

Torque	Group			*p* value			
	a: nicotine	b: nicotine+l-arg	c: no nicotine no l-arg	Total	a, b	a, c	b, c
Insertion	85.6 ± 17.5	44.4 ± 8.6	60.5 ± 9.1	0.025	0.020	0.194	0.497
Removal	79.87 ± 27.6	69.2 ± 37.8	107.5 ± 16.5	0.041	0.758	0.156	0.039
Change	− 5.7 ± 24.0	25.1 ± 37.8	47.0 ± 16.9	0.004	0.049	0.003	0.276
*p* value	0.521	0.103	< 0.001				

Data were presented as mean ± SD; column *p* value, extract from one-way ANOVA and Tukey post hoc; row *p* value, extract from paired *t* test

**Fig. 4:**
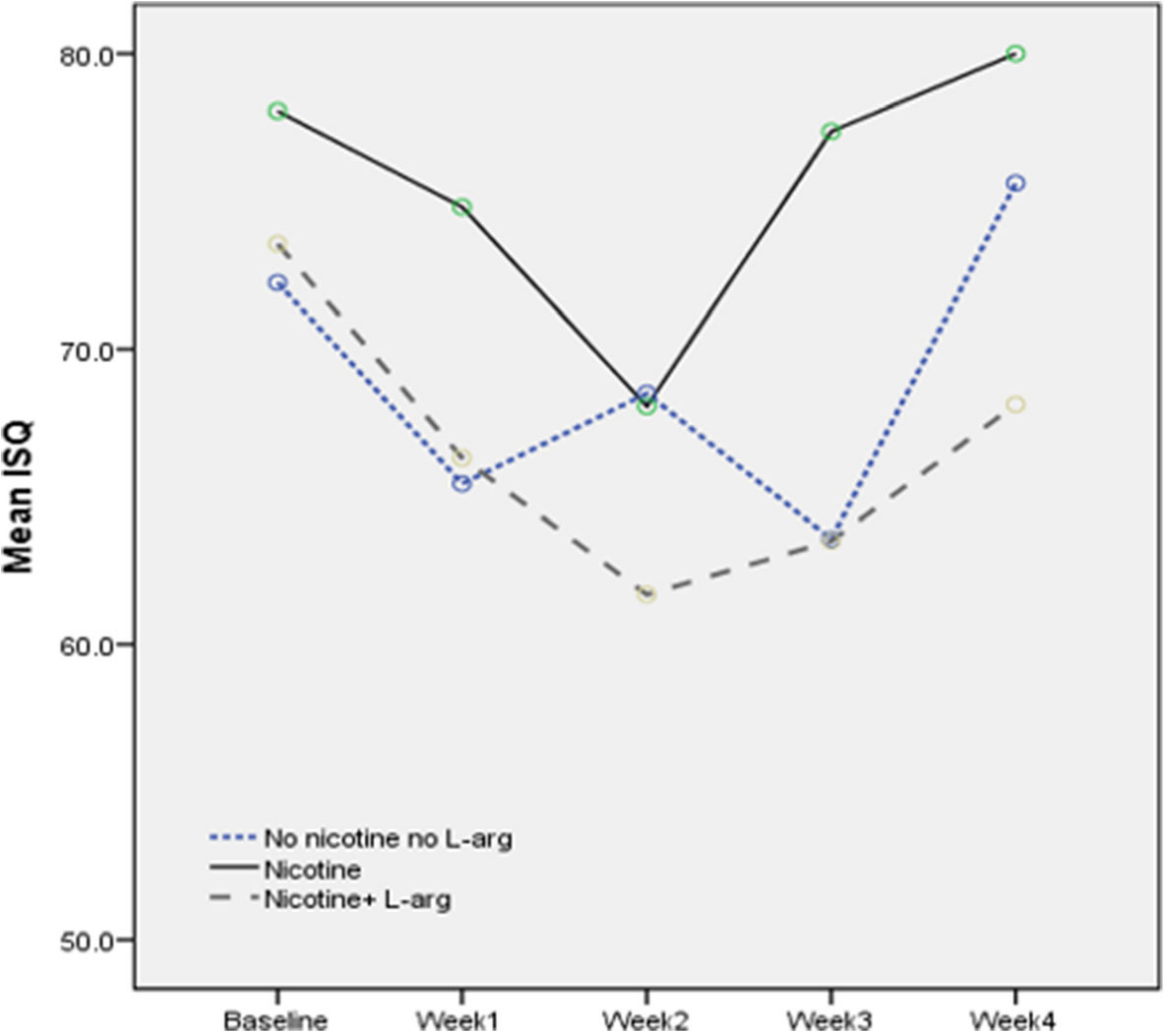
The mean ISQ was measured every week for 4 weeks in the three study groups

ISQ values are presented in Table 2. The results show that except in the first week, there are significant differences in ISQ between the groups in different periods. At the time of insertion, there were significant differences between the nicotine consumer group and nicotine+arginine consumer group (*p* = 0.009) and nicotine consumers group and placebo group (*p* = 0.001). In the first week, only nicotine consumers and placebo showed a significant difference (*p* = 0.048). In the third week, there was a significant difference between nicotine consumers compared to nicotine+arginine consumers (*p* = 0.003) and nicotine consumers compared to placebo (*p* = 0.003), but in the fourth week, there were only significant differences between the nicotine consumer group and the nicotine+arginine consumers (*p* = 0.009). ISQ in all of the groups has reduced at first but then increased over time. This process can be seen in all of the three groups (Fig. 4).

**Table 2:** Comparison of ISQ between the 3 groups in five different times

ISQ		Group			*p* value			
		a: nicotine	b: nicotine+l-arg	c: no nicotine no l-arg	Total	a, b	a, c	b, c
Baseline		78.0 ± 2.6	73.5 ± 2.3	72.2 ± 3.1	0.001	0.009	0.001	0.609
Week 1		74.8 ± 4.5	67.4 ± 8.3	65.4 ± 4.1	0.035	0.077	0.048	0.970
Week 2		68.0 ± 7.3	61.6 ± 6.7	68.5 ± 4.17	0.072	–	–	–
Week 3		77.3 ± 4.5	63.5 ± 4.9	63.5 ± 9.0	0.001	0.003	0.003	> 0.999
Week 4		80.0 ± 3.7	68.1 ± 9.5	75.6 ± 6.9	0.011	0.009	0.452	0.114
*p* value	Linear	0.158	0.213	0.616				
	Quadratic	< 0.001	0.016	< 0.001				

Data were presented as mean ± SD; column *p* value, extract from one-way ANOVA and Tukey post hoc; row *p* value, extract from repeated measure ANOVA

## Discussion

The present study tried to evaluate the effects of nicotine alone and in combination with arginine to find out any alterations in mechanical properties of early dental implant osseointegration in dogs’ mandible. The present study showed that nicotine could negatively affect the mechanical properties of the dental implant. Arginine partially compensated for the negative effect of nicotine. Nicotine alone or in combination with arginine resulted in inferior mechanical properties compared to placebo during 4 weeks of post-implantation.

Nicotine is known to reduce the proliferation of red blood cells, macrophages, and fibroblasts. It also affects tissue perfusion and healing by increasing platelet adhesion and vasoconstriction. Sympathomimetic action of nicotine stimulates epinephrine and norepinephrine release, which consequently causes vasoconstriction and limited tissue perfusion. Contemplating these effects, nicotine is expected to debilitate potential bone healing at the bone and implant interface. At 8 weeks, nicotine consumer rats showed that the bone matrix-related gene expression was downregulated, and bone formation around implants was decreased [20].

Berley et al. [21] found that bone-to-implant contact is decreased in rats receiving nicotine compared to control. Soares et al. showed a decrease in bone formation around hydroxyapatite implants placed in the tibia and femur of rats receiving nicotine compared to rats receiving water or alcohol [22]. On the other hand, Pereira et al. demonstrated that nicotine increases bone-forming enzymes’ synthesis and enhances osteoblasts’ growth and differentiation [23].

Cesar-Neto et al. found no difference in rats’ bone healing receiving or not receiving nicotine [24]. Balatsoaka et al. demonstrated an increase in bone density from 2 to 4 weeks around implants in the rabbit receiving nicotine [25]. These contradictory results might be due to cell cultures used and other methodological differences between these studies.

In the present study, nicotine has resulted in reduced removal torque compared to the placebo. Arginine supplementation has increased torque over 4 weeks, and there was no significant difference in removal torque between the nicotine and arginine consumer groups compared to placebo.

The bone around dental implants continues an active remodeling even after 5 years of implantation. Studies that investigated bone healing around dental implants after few weeks to 20 years or more implant functioning show that masticatory loads stimulate continuous remodeling after primary healing of an implant [6, 26]. The active remodeling is known to affect the bone quality, that is, the bone-implant contact area and the quality of the bone, which is influenced by the amount and distribution of collagen and minerals in the bone around dental implants. Thus, continuous remodeling contributes to implant mechanical stability over the functional years of a dental implant [4, 26, 27]. Although 4 weeks might be a short period in the present study, there was an increase in ISQ and removal torque values in all of the groups.

There is a strong correlation between the degree of tissue mineralization, as an indicator of bone quality, and mechanical properties of the bone [3, 28]. Incompletely mineralized newly formed bone due to bone remodeling adjacent to the implant is anticipated to be mechanically inferior to pre-existing bony tissues away from the implant. Therefore, In the present study, we have used mechanical measurements to evaluate the implant stabilities. Although histometric analysis would have given us more information, dental implants’ biomechanical behavior is one of the most important prognostic factors and an essential indicator of osseointegration.

Collagen is the dominant constituent of bony tissues that control the viscoelastic properties [3, 29, 30]. Under the load, protein fibrils of collagen show time-dependent deformation.

During the bone mineralization process, minerals are gradually arranged along the collagen fibrils and eventually create the mineral-collagen complex’s mechanical integrity. Thus, bone remodeling by changing the bone tissue composition alters bone’s mechanical properties such as viscoelastic characteristics—further studies are required to investigate bone’s viscoelastic properties around dental implants.

Many attempts have been made to find superior dental materials and growth agents to promote bone quality and quantity around and at the implant-bone interface. One of several agents is amino acids, which have been suggested that can hasten bone healing by enhancing local blood supply, stimulating growth factor, and increasing collagen synthesis [31].

In vitro studies have shown that amino acids such as arginine promote osteoblastic growth and differentiation by stimulating insulin growth factor-1 secretion [32–34]. Others found that arginine, lysine, and glycine are associated with collagen synthesis [10, 35]. Goel et al. [36] found that 2 g/day l-arginine increased bone mineral density by 11.6% in osteoporotic women for 2 years.

In the present study, arginine was supplemented to nicotine consumer dogs to help in the osseointegration of dental implants. Arginine supplementation’s beneficial effect might be due to arginine’s potential therapeutic effect on bone healing by enhancing nitrous oxide production and collagen synthesis. Cell proliferation precedes the synthetic phase of osteogenic bone-derived osteoblasts [10].

## Conclusion

Under the present study condition, arginine supplementation promotes bone healing and implant primary stability by improving dental implant osseointegration biomechanical characteristics. Further studies are required, paving the way for possible clinical application in the context of early and immediate implant loading.

## Data Availability

The datasets used and/or analyzed during the current study are available from the corresponding author on reasonable request.

## Abbreviations

ISQ: Implant stability quotient
ANOVA: One-way analysis of variance
